# Skeletal and non-skeletal factors associated with mandibular asymmetry in mixed dentition

**DOI:** 10.4317/jced.63704

**Published:** 2026-01-28

**Authors:** Silvana Cumpa-Dávila, Mariano Ortiz-Pizarro, Juan Carlos Julca-Lévano

**Affiliations:** 1 Facultad de Medicina, Universidad Católica Santo Toribio de Mogrovejo, Chiclayo, Peru

## Abstract

**Background:**

Mandibular asymmetry is a complex challenge during mixed dentition. Aim: To determine the skeletal and non-skeletal factors associated with mandibular asymmetry in patients with mixed dentition.

**Material and Methods:**

The observational and cross-sectional study included 271 digital panoramic and lateral skull radiographs of patients from radiographic centers. The Eagle.PS Plate Scanner program was used to evaluate condylar morphology, lower second molar development, lower first molar loss, cervical vertebrae maturation, gonial angle, vertical growth, cranial base angle, intermaxillary relationship, and asymmetry. Frequencies, Spearman's Rho coefficient, Chi-square, and binomial logistic regression were used.

**Results:**

57.1% of patients were female, 61.3% were aged between 6 and 9 years, and 30% were at CS4 stage. Mandibular body asymmetry is related to age ranges (p=0.011). Patients with increased vertical growth (p=0.037; OR=3.20) and loss of the lower first molar (p=0.016; OR=11.33) are more likely to have mandibular ramus asymmetry; however, patients with an open cranial base angle are less likely to have it (p=0.039; OR=0.25). On the other hand, patients with a closed gonial angle (p=0.024; OR=3.48) and a bird's beak mandibular condyle shape (p=0.046; OR=2.68) are more likely to have mandibular body asymmetry.

**Conclusions:**

Asymmetry in mandibular body is associated with the age, while a decreased gonial angle and a bird-beak condyle shape predispose to asymmetry in mandibular body height. An increased vertical growth pattern and the loss of the first permanent lower molar predispose to asymmetry in mandibular ramus height, while an increased cranial angle is associated with a lower probability.

## Introduction

Mandibular asymmetry is a frequent diagnostic challenge during mixed dentition, because at this stage there are discrepancies in the growth of the right and left sides of the jaw that can contribute to functional problems such as difficulties in chewing and jaw articulation, and can negatively affect facial harmony (1). The coexistence of primary and permanent teeth introduces variations inherent to tooth replacement that hinder clinical and radiographic interpretation, making early identification of growth abnormalities essential (1 , 2). Facial asymmetry, understood as the lack of balance between both sides of the face, can be caused by skeletal components or due to variations in soft tissues and occlusion. Clinically, it has been observed that differences in the height of the mandibular ramus and body are related to certain vertical morphological patterns of facial growth (2). A panoramic X-ray provides detailed information about the mandibular ramus and body, as well as the entire dentition (3). Habets et al. proposed an asymmetry index based on measurements of the mandibular body and ramus, classifying asymmetry as insignificant (0-2 mm), mild (2-3 mm), moderate (3-5 mm), and severe (&gt;5 mm) (4). These measurements are fundamental tools for the objective detection of asymmetries in growing patients (1). Determining skeletal maturity is equally important for establishing the appropriate timing of intervention. The cervical maturation method described by Baccetti evaluates the progressive morphological changes in the cervical vertebrae, which correlate closely with the intensity of mandibular growth (5 , 6). Among the skeletal factors frequently studied in asymmetry are the gonial angle (7), the vertical growth pattern usually determined by the SN.MP angle (8 , 9), variations in the cranial base angle, which modify the condylar position and mandibular length. Likewise, the maxillomandibular relationship influences growth balance and may be associated with bilateral differences in the mandible (10). In relation to non-skeletal factors, it has been reported that the development of the permanent lower second molar, as assessed by Nolla stages (11), and early loss of the lower first molar can lead to occlusal imbalances that favor the development of functional and subsequently structural asymmetries (12). Similarly, respiratory disorders, such as mouth breathing associated with Class II malocclusion, can alter cervical morphology and predispose to upper airway obstruction (13 , 14). Therefore, the aim of present study was to evaluate the skeletal and non-skeletal factors associated with height asymmetry in mandibular body and mandibular ramus during mixed dentition.

## Material and Methods

This cross-sectional observational study used an indirect observation technique on radiographic records and was approved by a research ethics committee through resolution No. 650-2024-USAT-FMED. The population consisted of digital radiographs of male or female patients with mixed dentition who attended an imaging diagnostic center with an indication for panoramic and cephalometric radiographs, while patients with incomplete imaging data and records that prevented measurement or interpretation were excluded. The sample size was determined using the formula for estimating a proportion within an infinite population. The calculation considered an asymmetry prevalence of 83.79% with respect to mandibular height reported by Ramírez-Yáñez (4), resulting in a minimum sample size of 217 patients with mixed dentition. Sampling was non-probabilistic, based on progressive access to the units of analysis. A team of three experts (A.A.N; E.L.G; J.C.J.L.) validated the content of a data collection form used to record variables. Subsequently, a pilot test was carried out with 30 units of analysis to evaluate the error of method in identifying points and measuring of angles and lines. As a result, according to Cohen's Kappa statistical analysis for nominal variables and Kendall's Tau-b for ordinal variables, a high level of agreement (r&gt;0.90) was obtained in inter-examiner and intra-examiner calibration. Subsequently, all radiographs were incorporated into the Eagle.ps Plate Scanner program (Dabi Atlante, São Paulo, Brazil), following the established criteria to carry out the corresponding analysis. Ten to twenty panoramic and cephalometric X-rays were examined each day, organizing the work into one- to two-hour sessions interspersed with 10- to 15-minute breaks to minimize visual fatigue. In the evaluation of panoramic radiographs, four variables were analyzed, such as the shape of the condyle observed in the upper part of the mandibular ramus, which was classified into four categories: type I (oval), type II (bird's beak), type III (diamond), and type IV (crooked finger) according to the morphology evident in the image (15). The stage of development of the second permanent lower molar was determined by applying Nolla's stages, observing the transition from radiolucent to radiopaque areas as mineralization progressed, until reaching stage 10, where the root and apical constriction showed complete mineralization (16). Finally, early loss of the first permanent lower molar was evaluated in the posterior region of the mandible; its presence was identified as a radiopaque image, while its absence was evident as a radiolucent area (17). Mandibular ramus asymmetry was assessed by drawing a line from the lowest point of the sigmoid notch to the angle of the mandible; while for the mandibular body, a line was drawn from the mesial point of the first permanent lower molar to the base of the mandible. These measurements were used in the Saglam index, which categorizes mandibular asymmetry as insignificant or no asymmetry (0-2.99%) and mild asymmetry (&gt;3%) (18), according to the following formula: (right value-left value) / (right value+left value) x 100. In the cephalometric analysis, the cervical vertebrae were traced according to Baccetti, and the second, third, and fourth cervical vertebrae were evaluated, where the presence or absence of concavity in the lower edge and the shape of the vertebrae, which were trapezoidal, square, horizontal rectangular, or vertical rectangular, were observed (6). As for the gonial angle variable according to the Björk-Jaraback analysis (19), it can be classified as normal (130 ± 7°), closed (&lt;123°), and open (&gt;137°). For this reason, the angle between the plane of the ramus, which was obtained by drawing a line connecting the articulare point (Ar), located at the intersection of the base of the skull with the posterior edge of the ascending ramus of the mandible, and the gonion point (Go), located at the inferior and posterior angle of the mandible, was measured. with the mandibular plane, which was determined by a line from the gonion point (Go) to the gnation point (Gn), corresponding to the most anterior and inferior point of the chin (20). On the other hand, the vertical growth pattern was evaluated and categorized as increased ( 38º), normal (26° - 38°), or decreased (26º) (8 , 9). Thus, the angle was obtained considering the S-N line, which runs from the Silla point (S), located in the sella turcica at the base of the skull, to the Nasion point (N), located at the junction of the frontal and nasal bones, with the mandibular plane mentioned above (21). To evaluate the skeletal relationship, the angle of the base of the skull was analyzed, determined by the N-S-Ba angle. This was obtained by drawing lines from the Nasion point (N) to the Silla point (S) and from this point to the Basion point (Ba). The reference values considered were normal (130°-135°), open (&gt;135°), and closed (&lt;130°) (22 , 23). For the ANB angle, two lines were drawn, one from the Nasion point (N) to point A, representing the location of the maxilla, and another connecting the Nasion point (N) to point B, indicating the position of the mandible (20). According to the parameters established by Steiner, the reference values were as follows: -1° to 5° corresponded to a Class I skeletal relationship, values greater than 5° indicated Class II, and values less than -1° defined Class III (24). -Statistical analysis The data obtained were recorded in a Microsoft Excel spreadsheet to encode the categorical responses into numerical codes, according to variables. Data analysis was performed using IBM SPSS software version 25 (Statistical Package for Social Sciences, SPSS Inc., Chicago, IL, USA). In the bivariate analysis, Spearman's Rho coefficient was used to correlate the covariates of age, sex, and cervical maturation status with ramus asymmetry and body asymmetry. The Chi-square test was used to evaluate the association between each factor and asymmetry. In the multivariate analysis, the research hypothesis was tested using binomial logistic regression to evaluate the joint interaction of skeletal and non-skeletal factors on the effect on ramus asymmetry and body asymmetry. The data for the analysis were presented as odds ratios (OR) and 95% confidence intervals, and the variables with the highest statistical significance according to the Wald test were included in the model. The hypothesis tests were two-tailed, with a confidence level of 95% and a significance level of 5%.

## Results

A total of 217 pairs of panoramic and cephalometric radiographs of patients in mixed dentition were evaluated. Most were female (57.1%), with a predominance of the 6-9 age range (61.3%), while the most frequent stage of maturation was SCM4 with 30% and the least frequent was SCM5 with 6%, as shown in Table 1.

Table 1

Table 2 shows that mandibular body height asymmetry is related to the age ranges of 6-9 years and 10-13 years (p=0.011).

Table 2

The rest of the variables were positively correlated with ramus and body asymmetry, but without statistical significance (p&gt;0.05). On the other hand, regarding the factors associated with mandibular ramus height asymmetry in patients with mixed dentition, those with an increased vertical growth pattern are 3.20 times more likely to present mandibular ramus height asymmetry compared to patients with normal vertical growth (p=0.037). Similarly, patients with absence of the permanent lower first molar were 11.33 times more likely to present mandibular ramus asymmetry compared to those with the first molar present (p=0.016). Conversely, patients with an increased cranial base angle are 0.25 times less likely to present mandibular ramus asymmetry compared to patients with a normal cranial angle (p=0.039). The model obtained has a conservative fit that explains 24.3% of the variance in mandibular ramus height asymmetry, as shown in Table 3.

Table 3

The other variables evaluated together with mandibular ramus asymmetry had no significant influence (p&gt;0.05). Table 4 shows the factors associated with mandibular body height asymmetry in mixed dentition, where patients with a decreased gonial angle are 3.48 times more likely to present mandibular body height asymmetry compared to patients with a normal gonial angle (p=0.024).

Table 4

Similarly, patients with a bird's beak mandibular condyle shape are 2.68 times more likely to present mandibular body height asymmetry compared to those with an oval condyle shape (p=0.046). The model obtained has a conservative fit that explains 20.8% of the variance in mandibular body height asymmetry. On the other hand, the other variables evaluated in the model did not contribute significantly to explaining mandibular body asymmetry (p&gt;0.05).

## Discussion

The aim of this study was to determine the skeletal and non-skeletal factors associated with mandibular asymmetry in patients with mixed dentition. It was observed that asymmetry in mandibular body height differed between patients in the first and second phases of mixed dentition, which is not the case with asymmetry in the ramus. This reinforces the results of studies that point to the mixed dentition period as a stage in which mandibular asymmetries can develop more frequently (4 , 11). In fact, studies have identified linear asymmetries in the mandibular ramus and body at an early age, suggesting that morphological discrepancies can be established from the initial stages of growth (7). The notable differences in growth on both sides of the jaw can be explained by the intense bone remodelling and functional adaptation that occurs during maxillofacial development, coupled with imbalances in tooth eruption or condylar growth (25). However, there are studies that indicate that asymmetry in height could be temporary, as the segments of the jaw adapt to each other to maintain functional balance. In addition, the functional demand that can produce a secondary adjustment of the angles through the remodelling process must be considered, even though not all jaw segments are regulated by the same growth and development mechanisms (4 , 11). Among the skeletal factors, it was found that an increased vertical growth pattern increased the likelihood of asymmetry in the height of the mandibular ramus. This result can be explained because a hyperdivergent pattern leads to downward and backward mandibular rotation, modifying the direction of condylar growth and favoring vertical differences between both sides (8). Previous research in children has reported that subjects with a dolichofacial tendency have a higher prevalence of asymmetries, probably due to less controlled vertical growth. The results reinforce this hypothesis by showing that the direction of facial growth directly influences mandibular morphology (25). On the other hand, it was observed that an open cranial base angle was associated with a lower probability of asymmetry in the mandibular ramus. This finding could be interpreted as a consequence of the more balanced arrangement of craniofacial structures when the cranial base is more extensive, allowing for more symmetrical mandibular development (26). However, this result differs from that reported in adults, where a lower mandibular ramus height has been described in hyperdivergent individuals, which is probably due to differences in selection criteria and the timing of bone growth, since in childhood the structures are still undergoing active remodeling (8). Regarding the mandibular body, the results showed that a closed gonial angle increased the probability of mandibular body asymmetry. This pattern could be due to the fact that a reduced angle reflects anterior mandibular rotation, which tends to manifest unevenly between the right and left sides due to functional or muscular variations (27). Vespasiano et al. (11) reported that the greatest asymmetry was found in the height of the ramus and the least in the gonial angle, which differs from the results; however, this difference could be due to not consider individual growth patterns, which significantly modify the expression of asymmetry (27). In fact, many of the disproportions in mandibular height are due to the different vertical positions in the gonial region, and even the craniofacial bones located in the upper facial regions, such as the maxilla, zygoma, and temporal bone, can play an important role in masking asymmetrical mandibular conditions due to the gonial angle (18). Likewise, it was found that the bird's beak condylar shape was associated with a higher probability of mandibular body asymmetry. This finding is due to the fact that condylar morphology reflects the type of functional adaptation experienced by the temporomandibular joint. A pointed or irregular shape, such as a bird's beak, could be related to greater unilateral remodeling, resulting from uneven loads or masticatory functions (28 , 29). Radiographic studies in children and adolescents have documented the presence of condylar shapes as frequent variants, so condylar morphology is proposed as a potential factor to be considered in conjunction with the skeletal pattern (13). Regarding non-skeletal factors, early loss of the first permanent lower molar showed a significant association with asymmetry in the mandibular ramus. This finding is explained by the fact that tooth loss alters the occlusal guide and favors compensatory tooth movements, generating asymmetrical functional loads that can affect mandibular growth. The literature describes that early loss of the first molar produces mesial migration, tooth inclinations, and changes in the molar relationship that can lead to functional and subsequently structural deviations (13 , 18 , 30). Among the limitations of the study is the use of two-dimensional images, which are widely employed in orthodontic diagnosis but do not allow for the evaluation of structures in all three planes of space. Furthermore, the study focused on a specific set of variables related to skeletal and non-skeletal factors. However, the inclusion of other variables, such as functional and muscular indicators or oral habits, could have provided a broader understanding of the elements involved in mandibular asymmetry. In addition, it is important to note that the study was conducted using non-probability sampling, and consequently, the results obtained cannot be generalized to other populations with mixed dentition.

## Conclusions

In patients with mixed dentition, significantly greater asymmetry in mandibular body height was observed radiographically as age increased. In this regard, patients with a decreased gonial angle and an irregular mandibular condyle shaped like a bird's beak are more likely to have asymmetry in mandibular body height. On the other hand, patients with mixed dentition with an increased vertical growth pattern and loss of the first permanent lower molar are more likely to have asymmetry in mandibular ramus height, while an increased cranial base angle could be a protective factor against this condition.

## Figures and Tables

**Table 1:** Table Age, sex, and stage of maturation of patients with mixed dentition.

Variable	Category	n	%
Age	6-9 years	133	61.3
	10-13 years	84	38.7
	Total	217	100
Sex	Female	124	57.1
	Male	93	42.9
	Total	217	100
Cervical maturation stage	CS1	34	15.7
	CS2	35	16.1
	CS3	54	24.9
	CS4	65	30.0
	CS5	13	6.0
	CS6	16	7.4
	Total	217	100

1

**Table 2:** Table Correlation coefficient of age, sex and maturation stage with mandibular height asymmetry.

Variable	Ramus asymmetry		Body asymmetry	
	Coefficient	p-value	Coefficient	p-value
Age	0.038	0.575	0.172	0.011*
Sex	0.050	0.460	0.013	0.852
Stage of maturation	0.003	0.963	0.080	0.189

(*) p-value: significance level ≤ 0.05. Spearman’s correlation coefficient

**Table 3:** Table Factors associated with asymmetry in mandibular ramus height in patients with mixed dentition.

Variables	Coefficient	p-value	OR	95% I.C. for OR	
				Lower	Upper
Sex, Male	-0.28	0.568	0.75	0.28	2.01
Age, 10-13 years	0.15	0.799	1.16	0.35	3.87
Maturation, CS2	-1.68	0.079	0.18	0.02	1.21
Maturation, CS3	0.48	0.495	1.63	0.40	6.65
Maturation, CS4	-0.23	0.768	0.79	0.16	3.77
Maturation, CS5	0.38	0.752	1.47	0.13	16.33
Maturation, CS6	0.95	0.387	2.58	0.30	22.31
Gonial angle, decreased	1.33	0.071	3.79	0.89	16.20
Gonial angle, increased	-0.03	0.981	0.96	0.05	16.45
Vertical growth, increased	1.16	0.037*	3.20	1.07	9.60
Vertical growth, decreased	-18.37	0.999	0.00	0.00	0.00
Class II	0.64	0.196	1.91	0.71	5.10
Class III	-0.03	0.983	0.96	0.05	16.72
Cranial base angle, increased	-1.35	0.039*	0.25	0.07	0.93
Cranial base angle, decreased	0.38	0.509	1.46	0.46	4.60
Condyle, bird's beak	0.45	0.474	1.56	0.45	5.38
Condyle, diamond	0.99	0.413	2.70	0.25	29.20
Condyle, twisted finger	-17.86	0.999	0.00	0.00	0.00
Second molar development, Nolla 4	21.45	1.000	SV	SV	IV
Second molar development, Nolla 5	20.23	1.000	SV	SV	IV
Second molar development, Nolla 6	19.26	1.000	SV	SV	IV
Second molar development, Nolla 7	18.61	1.000	SV	SV	IV
Second molar development, Nolla 8	19.65	1.000	SV	SV	IV
Second molar development, Nolla 9	19.03	1.000	SV	SV	IV
Second molar development, Nolla 10	19.64	1,000	SV	SV	IV
Upper pharyngeal pathways, reduced	0.48	0.730	1.63	0.10	26.15
Lower pharyngeal pathways, reduced	-0.58	0.295	0.55	0.18	1.66
First molar, premature loss	2.42	0.016*	11.33	1.58	81.06

(*) p-value: significance level < 0.05. Nagelkerke’s R-squared: 0.243

**Table 4:** Table Factors associated with asymmetry in mandibular height in patients with mixed dentition.

Variables	Coefficient	p-value	OR	95% I.C. for OR	
				Lower	Upper
Sex, Male	0.00	0.987	1.00	0.47	2.12
Age, 10-13 years	0,71	0.121	2.05	0.82	5.08
Maturation, CS2	-0.33	0.665	0.71	0.16	3.21
Maturation, CS3	0.54	0.405	1.72	0.47	6.18
Maturation, CS4	0.01	0.977	1.02	0.27	3.74
Maturation, CS5	-0.36	0.713	0.69	0.10	4.78
Maturation, CS6	0.06	0.939	1.07	0.18	6.33
Gonial angle, decreased	1.24	0.024*	3.48	1.18	10.25
Gonial angle, increased	1.54	0.198	4.70	0.44	49.76
Vertical growth, increased	0.67	0.122	1.96	0.83	4.59
Vertical growth, decreased	-20.12	0.999	0.00	0.00	0.00
Class II	0.32	0.429	1.37	0.62	3.04
Class III	1.68	0.159	5.38	0.51	56.00
Cranial base angle, increased	0.10	0.800	1.11	0.48	2.56
Cranial base angle, decreased	0.46	0.338	1.59	0.61	4.14
Condyle, bird's beak	0.98	0.046*	2.68	1.01	7.07
Condyle, diamond	0,88	0.360	2.42	0.36	16.15
Condyle, twisted finger	1.35	0.157	3.89	0.59	25.58
Second molar development, Nolla 4	19.08	1.000	SV	0.00	0.00
Second molar development, Nolla 5	17.77	1.000	SV	0.00	0.00
Second molar development, Nolla 6	18.68	1.000	SV	0.00	0.00
Second molar development, Nolla 7	18.39	1.000	SV	0.00	0.00
Second molar development, Nolla 8	19.07	1.000	SV	0.00	0.00
Second molar development, Nolla 9	18.96	1.000	SV	0.00	0.00
Second molar development, Nolla 10	18.67	1.000	SV	0.00	0.00
Upper pharyngeal pathways, reduced	0.09	0.937	1.09	0.10	11.01
Lower pharyngeal pathways, reduced	0.74	0.126	2.10	0.81	5.47
First molar, premature loss	-0.54	0.660	0.58	0.05	6.52

(*) p-value: significance level < 0.05. Nagelkerke’s R-squared: 0.208

## Data Availability

The datasets used and/or analyzed during the current study are available from the corresponding author.
